# Astrocyte Mechano-Activation by High-Rate Overpressure Involves Alterations in Structural and Junctional Proteins

**DOI:** 10.3389/fneur.2019.00099

**Published:** 2019-02-22

**Authors:** Nora Hlavac, Pamela J. VandeVord

**Affiliations:** ^1^Department of Biomedical Engineering and Mechanics, Virginia Polytechnic Institute, Blacksburg, VA, United States; ^2^Department of Research, Salem Veterans Affairs Medical Center, Salem, VA, United States

**Keywords:** astrocyte, overpressure, reactivity, cadherin, connexin, integrin, ICAM-1

## Abstract

Primary blast neurotrauma represents a unique injury paradigm characterized by high-rate overpressure effects on brain tissue. One major hallmark of blast neurotrauma is glial reactivity, notably prolonged astrocyte activation. This cellular response has been mainly defined in primary blast neurotrauma by increased intermediate filament expression. Because the intermediate filament networks physically interface with transmembrane proteins for junctional support, it was hypothesized that cell junction regulation is altered in the reactive phenotype as well. This would have implications for downstream transcriptional regulation via signal transduction pathways like nuclear factor kappa-light-chain-enhancer of activated B cells (NF-κB). Therefore, a custom high-rate overpressure simulator was built for *in vitro* testing using mechanical conditions based on intracranial pressure measurements in a rat model of blast neurotrauma. Primary rat astrocytes were exposed to isolated high-rate mechanical stimulation to study cell junction dynamics in relation to their mechano-activation. First, a time course for “classical” features of reactivity was devised by evaluation of glial fibrillary acidic protein (GFAP) and proliferating cell nuclear antigen (PCNA) expression. This was followed by gene and protein expression for both gap junction (connexins) and anchoring junction proteins (integrins and cadherins). Signal transduction analysis was carried out by nuclear localization of two molecules, NF-κB p65 and mitogen-activated protein kinase (MAPK) p38. Results indicated significant increases in connexin-43 expression and PCNA first at 24 h post-overpressure (*p* < 0.05), followed by structural reactivity (via increased GFAP, *p* < 0.05) corresponding to increased anchoring junction dynamics at 48 h post-overpressure (*p* < 0.05). Moreover, increased phosphorylation of focal adhesion kinase (FAK) was observed in addition to increased nuclear localization of both p65 and p38 (*p* < 0.05) during the period of structural reactivity. To evaluate the transcriptional activity of p65 in the nucleus, electrophoretic mobility shift assay was conducted for a binding site on the promoter region for intracellular adhesion molecule-1 (ICAM-1), an antagonist of tight junctions. A significant increase in the interaction of nuclear proteins with the NF-κB site on the ICAM-1 corresponded to increased gene and protein expression of ICAM-1 (*p* < 0.05). Altogether, these results indicate multiple targets and corresponding signaling pathways which involve cell junction dynamics in the mechano-activation of astrocytes following high-rate overpressure.

## Introduction

Traumatic brain injury (TBI) has proven particularly challenging to treat clinically because of the disparity amongst injury modes and severities. As such, there are no current FDA-approved treatments that exist for TBI. Many strategies have been employed against general hallmarks of cellular injury, such as inflammation and oxidative stress, however, with little success for recovery. There is a growing appreciation for the need to better characterize and understand how brain cells, individually and collectively, respond to mechanical damage. The pathological profile associated with mild TBI progression in particular, is quite complex and remains to be fully elucidated. It is especially critical to understand the potential differences in cellular and molecular hallmarks of TBI within the context of the mechanical injury itself. Importantly, the toxic environment associated with acute secondary injury acts as the initiator for prolonged neural cell dysfunction and cognitive deficits. This cascade of early cellular events offers the greatest promise for therapeutic intervention but is also highly complex, interdependent, and heterogeneous across TBI modes (1–5).

Although many of the same features of cellular stress exist across TBI modes, *in vitro* models have shown that brain cells have differential capacity to sense and respond to varied injury mechanics (6–9). This is important to consider in the context of high-rate injury scenarios, like blast neurotrauma, in which little is known about cellular tolerances. Blast neurotrauma represents a unique injury mode which has a high incidence rate in military populations exposed to explosive events (10, 11). From a mechanics standpoint, blast injury mechanisms are still largely controversial (12). Multiple proposed mechanisms from computational and experimental approaches exist and may include overpressure, shearing, and compression. These models have also suggested that shock waves generated by blast produces complex, high-speed pressure oscillations in brain tissue (13–15). This is important because hallmarks of cellular injury are dependent on overpressure mechanics (16–18), and behavioral aberrations seem to exist even at the lower injury thresholds (19–22).

One of the prominent secondary features of central nervous system (CNS) trauma is glial reactivity. Both microglia and astrocytes play a significant role in mediating the progression of secondary damage. Astrocytes, in particular, are multi-functional cells that act in the healthy brain to maintain ionic and trophic support for neurons as well as serve in active roles for cognitive functions (23–27). Astrocytes have emerged as a promising therapeutic target in TBI because of their diverse roles in metabolic and ionic homeostasis, structural integrity and tissue repair (28–30). This is especially true when considering their potential to communicate and adequately respond to injured neurons in a myriad of CNS insults. Specifically, impaired neuronal-astrocytic signaling can lead to excitotoxicity, metabolic failure and neurodegeneration, all of which have implications for the memory deficits and behavioral outcomes of TBI (31, 32).

Astrocyte “classical” reactivity is ubiquitously characterized by altered expression of intermediate filament protein expression, such as glial fibrillary acidic protein (GFAP), and by increased proliferation (28, 33). Astrocyte reactivity has been well characterized following *in vivo* blast TBI, and most notably involves classical reactivity with increased GFAP expression in astrocytes (16, 34–37). Studies *in vitro* have shown that even in the absence of other cell types, astrocytes assume an activated phenotype in response to varied mechanical perturbations (38, 39). There is strong evidence from *in vitro* studies to elude to a mechanical basis for disruption and reactivity of astrocytes (6–8, 40–42). Although blast-relevant (i.e., high-rate) *in vitro* models for brain cell reactivity are mostly recent in development, they have also shown that brain cells are differentially susceptible to insult at higher rates (43–45).

Of particular interest in this study is deciphering changes in regulation and expression of multiple classes of cellular junction proteins as they may relate to classical features of astrocyte reactivity following high-rate overpressure. The regulation of these molecules is important not only for understanding changes to cell phenotype but also to signal regulation and cell function. More specifically, there is a need to understand dynamic changes in astrocyte network communication as a means for autologous phenotypic activation and regulation (46). There are three major classes of cell junction proteins: gap junctions, anchoring junctions, and tight junctions. Gap junctions, notably connexins (CX), form dimers between cells to allow for passage of small molecules like ATP and other secondary messengers. Anchoring junctions form between cells and with the surrounding extracellular matrix (ECM). These proteins are necessary for maintaining cell shape and motility as well as structural integrity of tissue. Adaptor proteins such as vinculin act to connect the anchoring proteins to the cytoskeleton and create a force sensing mechanism for the whole cell. Lastly, tight junctions form between two cell membranes to prevent the passage of molecules between their spaces. Upon mechano-stimulation, adhesion signaling is activated via phosphorylation of a broad class of kinases, including focal adhesion kinase (FAK), which together direct numerous cell behaviors (47). Downstream of these adhesion complexes, signals diverge on several major pathways, including the mitogen-activated protein kinase (MAPK) and nuclear factor kappa-light-chain-enhancer of activated B cells (NF-κB) pathways. Together, these transduction pathways have important influence on astrocyte phenotype after injury as they broadly modulate cell survival, activation, migration, and differentiation, among other functions (46, 48, 49).

Altogether, adhesion and signal transduction molecules have broad influence on cell function and phenotype and therefore may be important modulators of astrocyte reactivity and network function. Motivation for this work has arisen from studies on other cell types which have extensively shown mechano-stimulation signaling responses and phenotypic shifts as a result of transient and prolonged mechanical stress. Although biochemical signals also activate signal transduction pathways, several recent studies have explored how adhesion signaling and mechano-stimulation elicit cellular reactivity in various contexts (50–53). This coincides with evidence suggesting astrocytes are responsive to isolated mechanical insult (54, 55). Studies have suggested a role for mechanobiological cues and changes in the extracellular environment of injured brain in damage cascades which may be closely coupled with adhesion properties (56–60).

The following work combined the study of biological cues related to cellular adhesion with well-documented aspects of astrocyte reactivity in an effort to understand how isolated high-rate overpressure may contribute to particular astrocyte phenotypes. While certain aspects of astrocyte reactivity are ubiquitous across injury modes, others are highly dependent on injury severity, insult mechanics, and location within the brain, among other factors. Therefore, it is possible that reactive phenotypic features are controlled by complex and interconnected injury mechanisms. There is a need for methodologies to study precise mechanisms of cellular responses, particularly to high-rate insults. This hypothesis was explored through the use of a novel *in vitro* high-rate injury device developed based on a study conducted on intracranial pressure profiles in rodents exposed blast neurotrauma (13). The device allows for controlled and repeatable cellular exposure to compressive-type overpressure in an effort to provide a means to better understand the mechanical basis for brain cell responses to high-rate injury.

## Materials and Methods

### Primary Astrocyte Cultures

**Figure 1:**
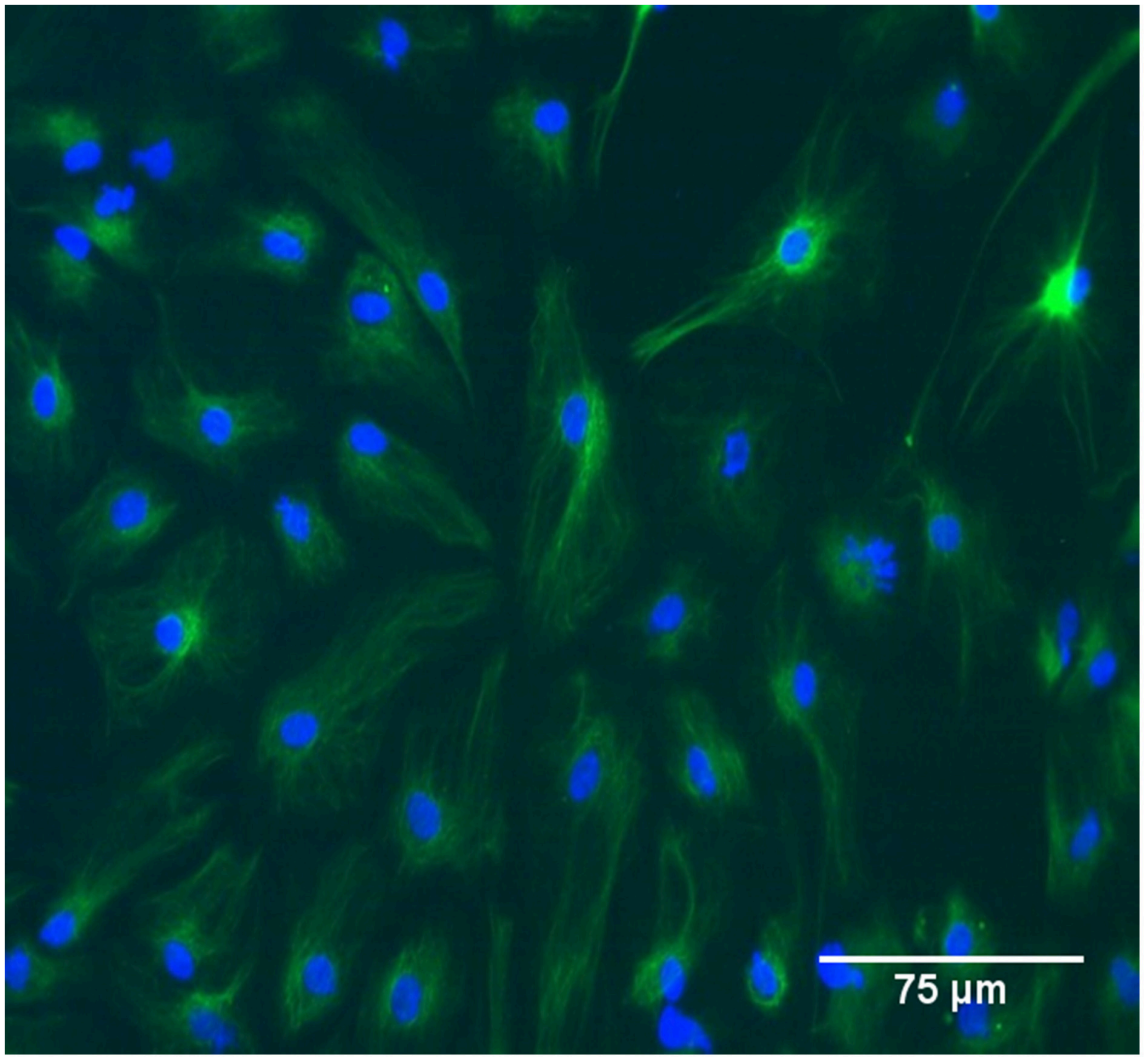
The study population was GFAP-positive primary rat astrocytes between passage zero and four. In the image cells were immunolabeled with GFAP and tagged with FITC. Scale bar is 75 μm.

Brain cortices were isolated from P2 Sprague-Dawley rat pups in accordance with protocols approved by Virginia Tech's Institutional Animal Care and Use Committee. Cortical tissue was enzymatically digested in 0.05% trypsin for 5–10 min and cultured up to 14 days to allow populations to reach confluence before initial passage. Seven days after isolation, other resident cells were mechanically removed from cultures by shaking for 24–48 h. Cells were maintained in Dulbecco's modified Eagle's medium (DMEM/F12, Gibco Cat# 11320) supplemented with 10% fetal bovine serum and 1% antibiotic-antimycotic (Gibco Cat# 15240-062). Cultures were routinely stained with anti-GFAP (Abcam Cat# ab7260, RRID: AB_305808) to ensure selection of a homogeneous population of astrocytes for this study (Figure 1). Prior to testing, astrocyte monolayers were seeded in standard six-well plates at a density of 1 × 10^5^ cells per well and were cultured for 6–7 days.

### High-Rate Overpressure Simulator (HOS) for *in vitro* Mechanical Exposure

**Figure 2:**
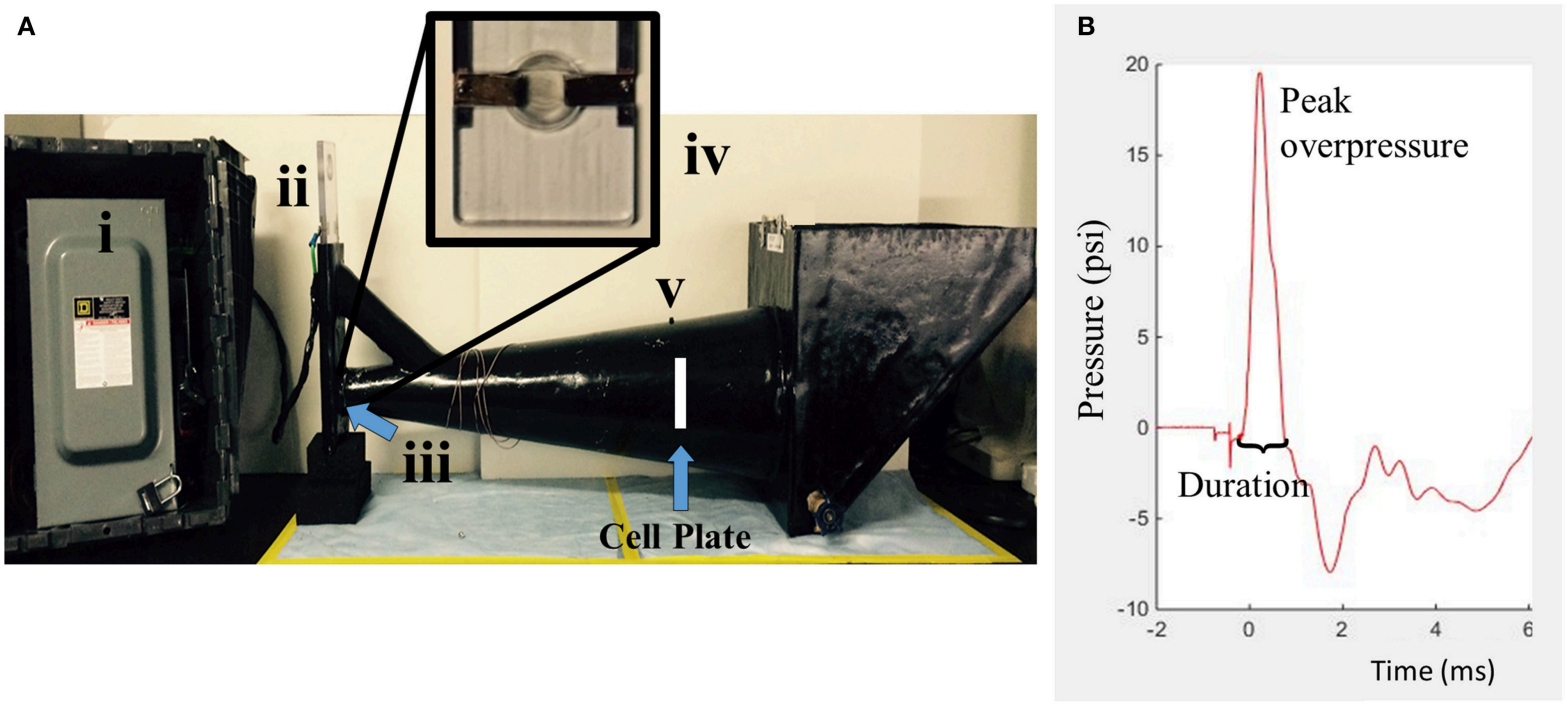
The HOS is a water-filled chamber which exposes *in vitro* samples to high-rate overpressure via an exploding bridge wire mechanism. **(A)** The generator is composed of four main parts. (i) Capacitor (ii) Circuit mechanism (iii) Wire bridge (inside) (iv) Wire bridge (zoomed) (v) Piezo transducer. **(B)** The simulated high-rate overpressure profile is designed to mimic intracranial pressure traces from *in vivo* blast testing.

Upon reaching confluence, cell cultures were exposed to isolated, transient overpressure using a custom underwater HOS (Figure 2). The chamber was designed following a study of the intracranial pressure profiles associated with primary blast exposure in rodents in which pressure inside the brain mimicked and amplified the high-rate compression wave (13). The HOS is a one-chamber conical device that creates repeatable high-rate overpressure through an exploding bridge wire technique (61, 62). This mechanism operates by charging a closed electrical circuit which contains a small bridge component. The bridge consists of two angled plates over which a thin wire is tightly suspended. This portion of the circuit is submerged within the HOS as denoted in Figure 2. Upon discharging the attached capacitor, current flows through the circuit to the point of least resistance (at the bridge). The bridge wire is subsequently vaporized upon high current passage and produces a repeatable high-rate compression wave. For testing, the chamber was filled with warmed reverse osmosis water (at 37°C). Cell plates were filled completely with culture medium (no added serum) and sealed with sterile parafilm. The cells were then placed in a holder such that the well plate was perpendicular to the flow field upon wave propagation. The HOS was instrumented with a piezo transducer (Meggitt Cat# 8350C or PCB Cat# 113B21) located in the wall of the chamber directly adjacent to the cell cultures. Cells were exposed to an average positive overpressure of 20 psi (138 kPa) with a 1 ms positive duration. This system was particularly advantageous for the study of high-rate overpressure, or blast-like injuries, because cells were exposed directly to the propagating compression wave with little to no impedance change as it was conducted in a completely submerged environment. Sham samples paralleled each overpressure-exposed plate and underwent the same preparation and placement in the HOS, without exposure to overpressure.

### RNA and Total Protein Extraction

In order to analyze molecular alterations related to cellular reactivity, adhesion and signaling, astrocyte RNA and total protein were collected at time points between 24 and 72 h after overpressure exposure. After removing culture medium, Trizol reagent (ThermoFisher Cat# 15596018) was incubated on samples at room temperature. The remainder of the protocol was based on manufacturers' recommendations. Following phase separation using chloroform and centrifugation, RNA was isolated and precipitated using propanol, washed with ethanol, dried and resuspended in water. MinElute (Qiagen Cat# 74106) spin columns were used under manufacturer's protocol to purify RNA samples. DNA contamination was removed using DNase treatment (Promega Cat# M6101) at 37°C for 30 min. Samples were quantified using spectrophotometry, and those with 260/280 ratios between 1.8 and 2 were used for further analysis by polymerase chain reaction (PCR).

Proteins were extracted from the phenol phase using ethanol and propanol followed by three 20 min washes with 0.3 M guanidine hydrochloride in 95% ethanol and one 20 min wash in ethanol. Samples were re-suspended in 1:1 mixture of 1% sodium dodecyl sulfate solution and 8 M urea in 1 M Tris-HCl (pH = 8.0) and protease inhibitor (Sigma-Aldrich Cat# P8340) at 1:100. Protein samples were homogenized for 10 s followed by a 10 min incubation at 55°C to facilitate resuspension. This process was repeated two more times. Alternatively, some analyses involved protein isolation by a standard lysis protocol. Instead of Trizol, cells were lysed in a buffer containing 40 mM Tris-HCl (pH = 7.5), 150 mM NaCl, 2.5 mM EDTA and 1% Triton X-100. After scraping cells from plate surface, samples were placed on ice and shaken vigorously (~600 rpm) for 30 min. Following centrifugation at 16,000 × g for 20 min, the solubilized proteins were transferred for further applications. Total protein samples were quantified by BCA assay (Pierce Cat# 23225) for use in Western blotting experiments described below.

### Reverse Transcription Real-Time PCR (qPCR)

Complementary DNA was synthesized from 1 μg of RNA by incubation with random hexamers and equimolar (10 mM) deoxynucleotide solution with dATP, dCTP, dGTP, and dTTP. Reverse transcriptase M-MLV (Invitrogen Cat# 28025-013) was added to convert RNA to cDNA. A qPCR master mix was prepared using SYBR green, ultrapure water, and primers at a final concentration of 0.33 M forward and 0.33 M reverse. Primers were designed using PrimerExpress and are listed in Table 1. Analysis of gene expression was conducted using a delta-Ct method with glyceraldehyde 3-phosphate dehydrogenase (GAPDH) as a housekeeping gene. Results are shown as normalized gene expression relative to the sham average for each gene (i.e., sham = 1).

**Table 1:** Gene sequences from PrimerExpress used for qPCR analysis.

**Gene**	**Abbrev**.	**Forward sequence**	**Reverse sequence**
Connexin-43	CX43	TACAGCGCAGAGCAAAATCG	GGCGTGCGAGTTGGAGAT
Intercellular adhesion molecule	ICAM-1	GACAGTGCTGTACCATGATCAGAAT	CCCGCAATGATCAGTACCAA
Integrin beta-1	Int β1	GAAGAGTCTTGGGACGGATCTG	GCCAATGCGGAAGTCTGAA
Vinculin	Vinc	TCCTGCGCGGGATTACC	CAGACGTTCCAGAGAGGATTCC
Glyceraldehyde 3-phosphate dehydrogenase	GAPDH	TGGCCTTCCGTGTTCCTACC	AGCCCAGGATGCCCTTTAGTG

### Western Blotting

A capillary-based automatic western blotting system called Wes (Protein Simple) was used for relative protein quantification. Supplies for the assays were purchased from Protein Simple and include separation modules (Cat# SW-004), anti-mouse detection modules (Cat# DM-002) and anti-rabbit detection modules (Cat# DM-001). Samples were prepared by following the manufacturer's protocol. This included a 10 min reducing step at 95°C with DTT (supplied). Primary antibodies used to probe total protein samples were anti-GFAP (Abcam Cat# ab7260, RRID: AB_305808), anti-CX43 (Novus Cat# NB100-91717, RRID: AB_1216521), anti-N-cadherin (Novus Cat# NBP1-48309, RRID: AB_10011059), anti-ICAM-1 (Novus Biologicals, NBP2-22541), anti-proliferating cell nuclear antigen (PCNA, Cell Signaling Technology Cat# 13110, RRID: AB_2636979), integrin-β1 (Cell Signaling, D6S1W), phospho-FAK (Y397, Abcam Cat# ab81298, RRID: AB_1640500), anti-p38 (Cell Signaling Technology Cat# 8690, RRID: AB_10999090), and anti-p65 (Cell Signaling Technology Cat# 8242, RRID: AB_10859369). Loading controls used in this study were either anti-β-actin (Sigma-Aldrich Cat# A5441, RRID: AB_476744) or anti-GAPDH (Novus Cat# NB600-502, RRID: AB_10077682). Secondary antibodies and other reagents were all supplied through the manufacturer. Standard settings of 25 min for separation time (at 375 V), 5 min for blocking, 30 min for primary antibody incubation, and 30 min for secondary antibody incubation were maintained for all experiments. Prior to experimentation, each antibody was optimized with samples to ensure a dynamic linear range for signals. Compass for SW software v.3.1 (Protein Simple) was used to quantify protein levels from area measurements derived from electropherograms at the same exposure time (5 or 15 s depending on the protein target) across all data plates. The software calculates the area under the curve using standard peak fits for all samples (Figure S1). Target proteins were first normalized to their respective loading controls and then to the overall sham average. Results are displayed as normalized expression relative to sham averages. It should be noted that the bands shown in the figures are digitized, clipped forms of these electropherograms which are meant to be representative of how the proteins traveled through the capillary system on the Wes and for qualitative visual comparison.

### 3-(4,5-Dimethylthiazol-2-yl)-2,5-Diphenyltetrazolium Bromide (MTT) Assay

Following *in vitro* overpressure exposure, astrocytes were assessed for metabolic (NADPH-dependent) activity by MTT assay. At 24 and 48 h post-overpressure, cell media was changed and supplemented with tetrazolium dye (Sigma-Aldrich Cat# M2128) at a final concentration of 0.25 mg/mL. Cultures were incubated at 37°C for 3 h with the tetrazolium before being dissolved in dimethyl sulfoxide. Samples were then transferred in triplicate to a 96-well plate and absorbance was read at 570 and 650 nm (background). Percent activity was calculated based on average optical density measurements for the sham samples.

### Nuclear Protein Extraction

Nuclear proteins were isolated using a commercially available kit from Epigentek (Cat# OP-0002-1). Manufacturer's protocols were followed. Briefly, cells were trypsinized and centrifuged for 5 min at 1,000 rpm. Cell pellets were incubated with lysis buffer NE1 (kit component) for 10 min on ice. Following, samples were vortexed at 11,000 × g for 1 min, with the supernatant containing the cytoplasmic proteins. The nuclear protein pellet was then incubated with NE2 buffer (kit) and repeatedly vortexed to re-solubilize. Proteins were quantified using a microBCA assay (Pierce Cat# 23235) for use in Western blotting experiments.

### Electrophoretic Mobility Shift Assay (EMSA)

EMSA is a gel-based assay for the study of DNA-protein interactions. Nuclear proteins were extracted as described above from samples collected at 48 h post-exposure or sham treatment. Oligonucleotides were constructed based on the NF-κB binding site at −218 on the rat ICAM-1 promoter. The 45-mer used for this study began at −236 and contained an NF-κB consensus sequence (5′-GGAAATTCC-3′). Biotinylated and unlabeled sequences were purchased from Integrated DNA Technologies. Reactions were carried out in accordance with manufacturer's protocol for LightShift Chemiluminescent EMSA kit (Pierce Cat# 20148). Sample preparation included recommended volumes of binding buffer, poly (dI·dC), 50% glycerol, 1% NP-40, and 100 mM MgCl_2_. Biotinylated DNA concentrations (200 nmol) were optimized to ensure a linear region for reaction detection. Nuclear proteins were diluted to equal total concentration with a final reaction concentration of 120 ug/mL in DNA mixtures. Samples were run on 6% DNA retarding gels (Invitrogen Cat# EC6365BOX) and transferred onto 0.45 μm nylon membranes (Biodyne Cat# 77016) for detection on a FujiFilm LAS-3000 CCD camera. In order to determine specificity of binding, several experiments were conducted with unlabeled sequence in excess to ensure changes chemiluminescence.

### Immunohistochemistry

Cell samples were fixed with ice-cold methanol for 7 min at −20°C. Following fixation, samples were permeabilized for 15 min with PBS containing 0.5% Triton X-100, followed by 2 min with PBS containing 0.01% Tween-20. Hydrochloric acid was used to depurinate samples for 30 min. Next, samples were incubated with PBS containing 0.01% Tween-20 for 10 min and then were blocked with 10% bovine serum albumin for 1 h. Overnight incubation with 5-methylcytosine (Epigentek Cat# A-1014-100) was conducted at 4°C. Secondary antibody was incubated on the samples for 1.5 h. Images were obtained using a standard fluorescent microscope (Zeiss) with a 20× objective.

### Statistics

Statistical comparisons were conducted between groups using JMP software v13.0.0 (SAS, Virginia Tech). ANOVA was used to analyze significant differences amongst groups, followed by Student's *t*-test for individual group comparisons. The assumptions for normality and homoscedasticity were confirmed by Shapiro-Wilk and Levene's tests, respectively. In the event that data was not normal, a logarithmic transformation was performed to conduct statistical comparisons (GFAP data, Figure 2). For sample sets with unequal variances, a Welch's *t*-test was performed (CX43 data, **Figure 5** and nuclear data, **Figure 6B**). Statistical outliers were determined using residual analysis, and a *p* < 0.05 was considered significant. Total number of replicates are denoted as the variable “n.” These represent individual replicates. Biological replicates were considered as blocks because they were seeded as entire six-well plates of overpressure/sham tests and were from the same line of cells (from the same animal and passage number). Three to four sample blocks were used per time point depending on application with three to four individual replicates from each block.

## Results

### High-Rate Mechanical Insult Induces Multiple Hallmarks of Astrocyte Reactivity

**Figure 3:**
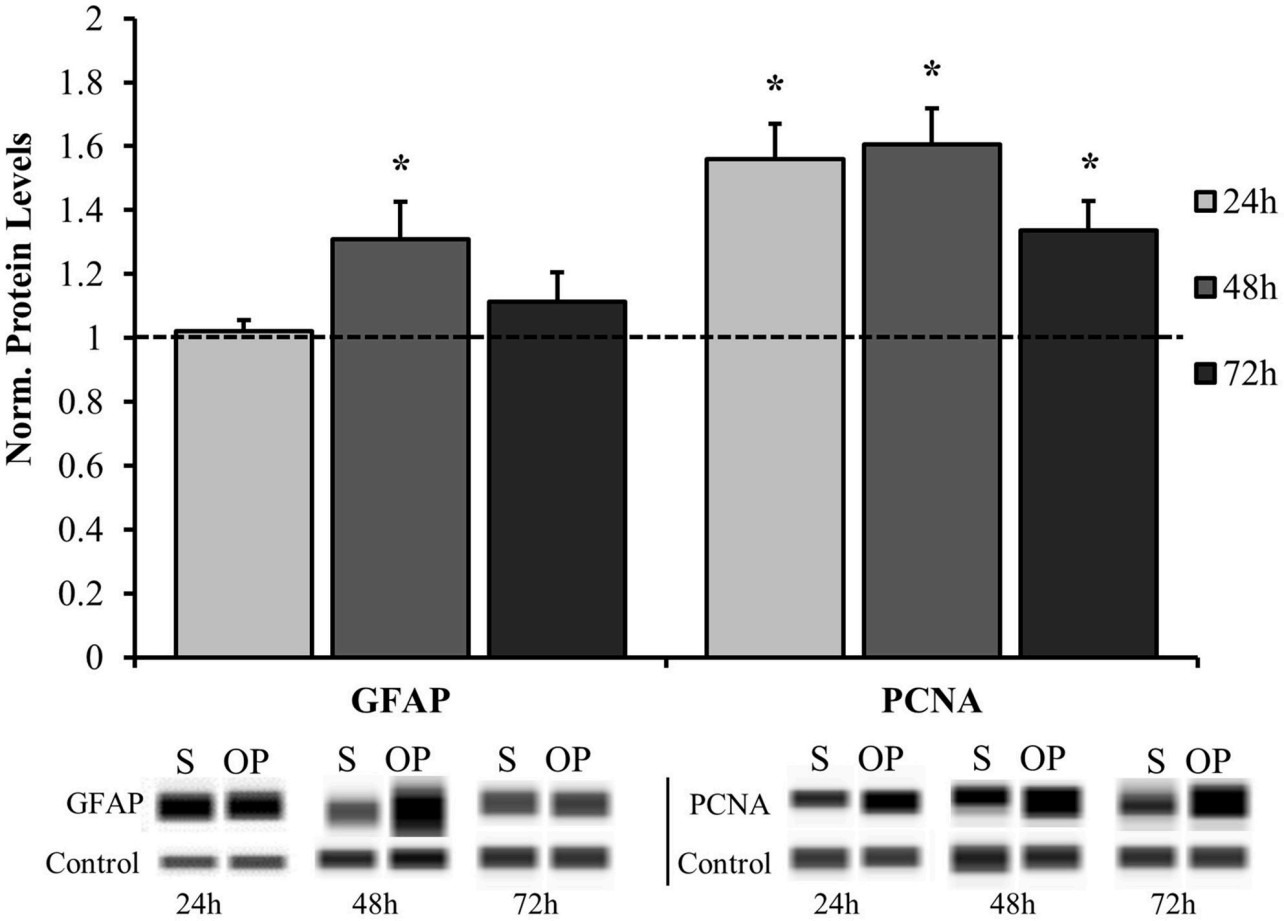
Time course analysis for normalized protein expression of classical reactivity markers. GFAP was significantly elevated in the overpressure group at 48 h while PCNA was increased at 24, 48, and 72 h compared to their respective shams. ^*^p < 0.05, Data are represented as mean ± SEM, *n* = 8–13/group.

**Figure 4:**
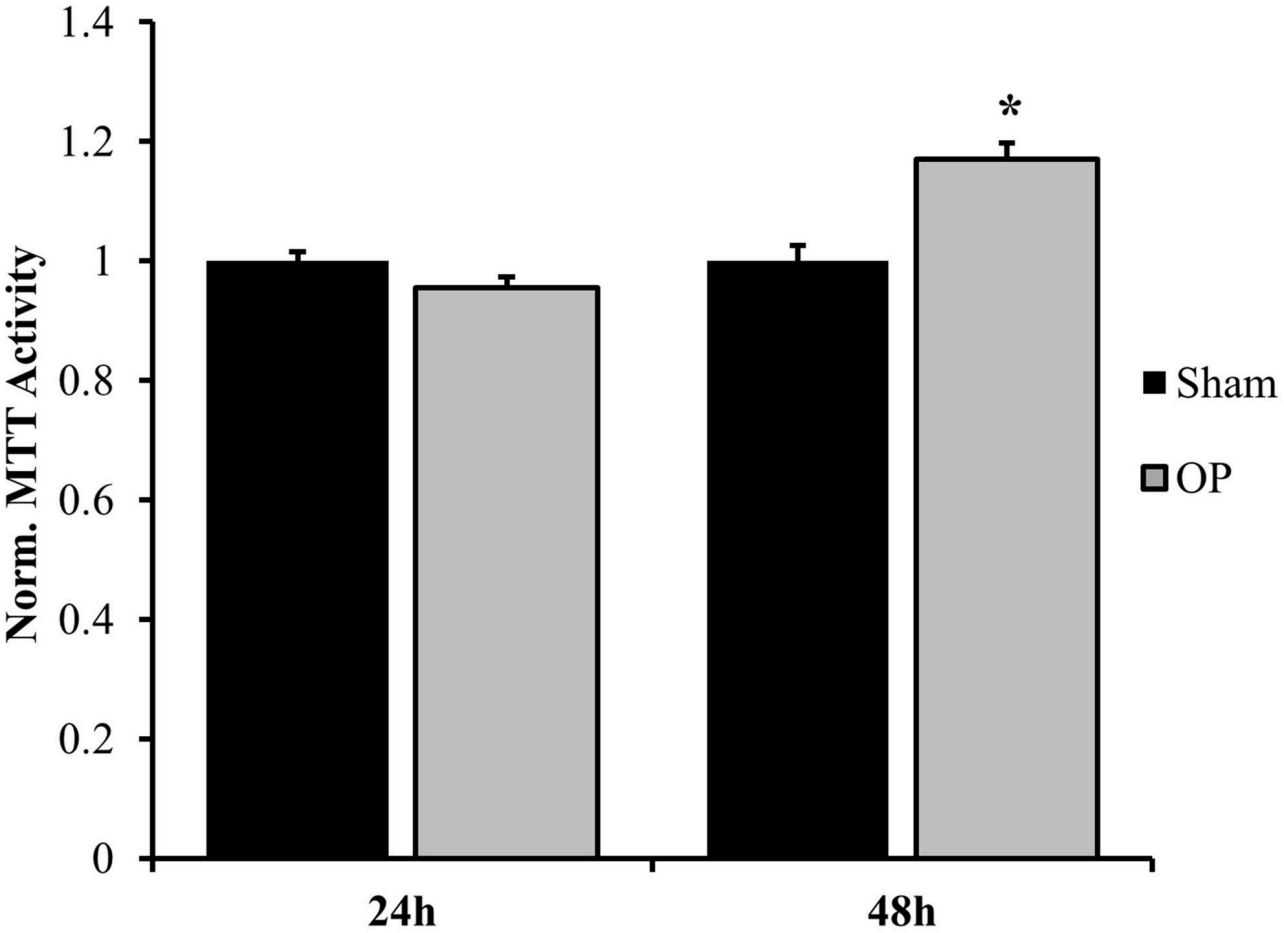
Normalized MTT metabolic activity. A significant change was observed at 48 h after overpressure exposure as compared to sham. ^*^p < 0.05, Data are represented as mean ± SEM, *n* = 8–9/group.

The results herein focus astrocyte reactivity to the mechanical perturbation of a high-rate compression wave in the absence of other cellular signals. The first objective was to understand the extent and time course of reactivity induced by mechano-activation of astrocytes. Samples were exposed to overpressure parameters summarized in Table 2. The target overpressure mimics intracranial pressures traces based on a rodent model of low severity blast neurotrauma (13, 16, 36). To assess the reactive phenotype, protein levels for PCNA and GFAP were at 24, 48, and 72 h post-exposure by Western blot analysis. Figure 3 shows that astrocytes assumed a proliferative/reparative phenotype as measured by PCNA at 24 h post-exposure which was sustained throughout the final 72 h time point assessed. Each overpressure-exposed group was statistically different from its respective sham at the same time point (*p* < 0.05). It should be noted that despite increased PCNA, there were no detectable changes in MTT metabolism until 48 h at which point there was a significant increase in enzymatic activity (*p* = 0.0005; Figure 4) Otherwise, GFAP expression had a delayed increase at 48 h post high-rate overpressure exposure. These results are consistent with a previous study on gene expression of GFAP in C6 glioma cells exposed to the same mechanics (62). Increased GFAP in conjunction with proliferative/reparative potential will be referred to as “classical reactivity.” These data establish a time course at which to evaluate the molecular phenotype of mechanically-activated astrocytes using an *in vitro* high-rate overpressure model. This transition in phenotype which occurred between the 24 and 48 h time points will be the focus for the subsequent analyses.

**Table 2:** Summary of shock wave parameters.

	**Avg ± Std dev**
Peak overpressure [psi]	19.9 ± 5.1
Positive peak duration [ms]	0.95 ± 0.28
Total tests, *n* = 25	

### Cell Junction Molecules Become Dysregulated in Reactive Astrocytes

**Figure 5:**
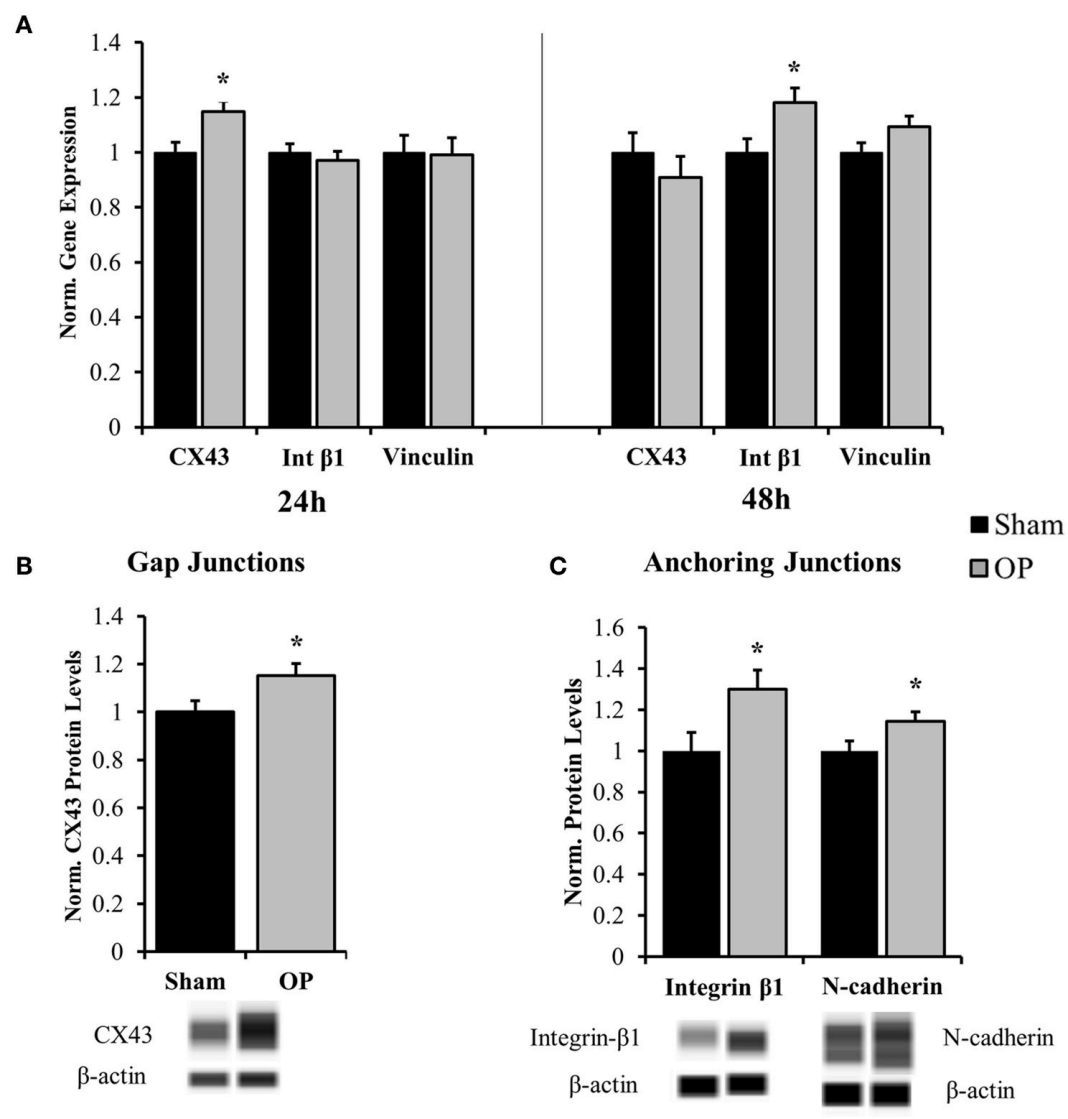
Gene and protein expression for cell junction targets. **(A)** Gene expression analysis showed increased gap junction (CX43) regulation at 24 h after high-rate overpressure and increased anchoring junction (integrin) regulation at 48 h as compared to sham. No changes were observed in vinculin mRNA expression. **(B)** Western blot results confirmed increased protein levels of CX43 at 24 h after overpressure as compared to sham. **(C)** Anchoring junction proteins integrin-β1 and N-cadherin were significantly elevated as compared to sham at 48 h after overpressure. ^*^p < 0.05, Data are represented as mean ± SEM, gene: *n* = 7–9/group, protein: *n* = 11–12/group.

Several classes of junctional molecules were assessed for this study. Gene expression analysis was conducted for one gap junctional target (CX43), one anchoring junction target (integrin-β1), and one intermediate protein (vinculin). PCR results determined that gap-junctional CX43 was upregulated compared to sham at 24 h (*p* = 0.028), prior to the anchoring junction protein integrin-β1 (Figure 5A). As a gap junctional protein, CX43 mainly functions as a signaling protein for small molecule transport. Integrin proteins are associated with cell-matrix and cell-cell contact and are coupled with cytoskeletal elements. Integrin-β1 was upregulated as compared to sham in conjunction with structural reactivity at 48 h (*p* = 0.025). Conversely there was no significant difference in expression of the adaptor protein vinculin (*p* = 0.092 at 48 h). Subsequent protein analysis by Western blot confirmed that increased gene expression of CX43 at 24 h (*p* = 0.035, Figure 5B) and integrin-β1 at 48 h (*p* = 0.029, Figure 5C) corresponded to increased protein translation after overpressure. Moreover, another anchoring junction protein, N-cadherin, was concurrently increased at 48 h relative to sham as assessed by Western blot (*p* = 0.047, Figure 5C).

### Astrocytes Undergo Delayed Signal Transduction in Response to High-Rate Insult

**Figure 6:**
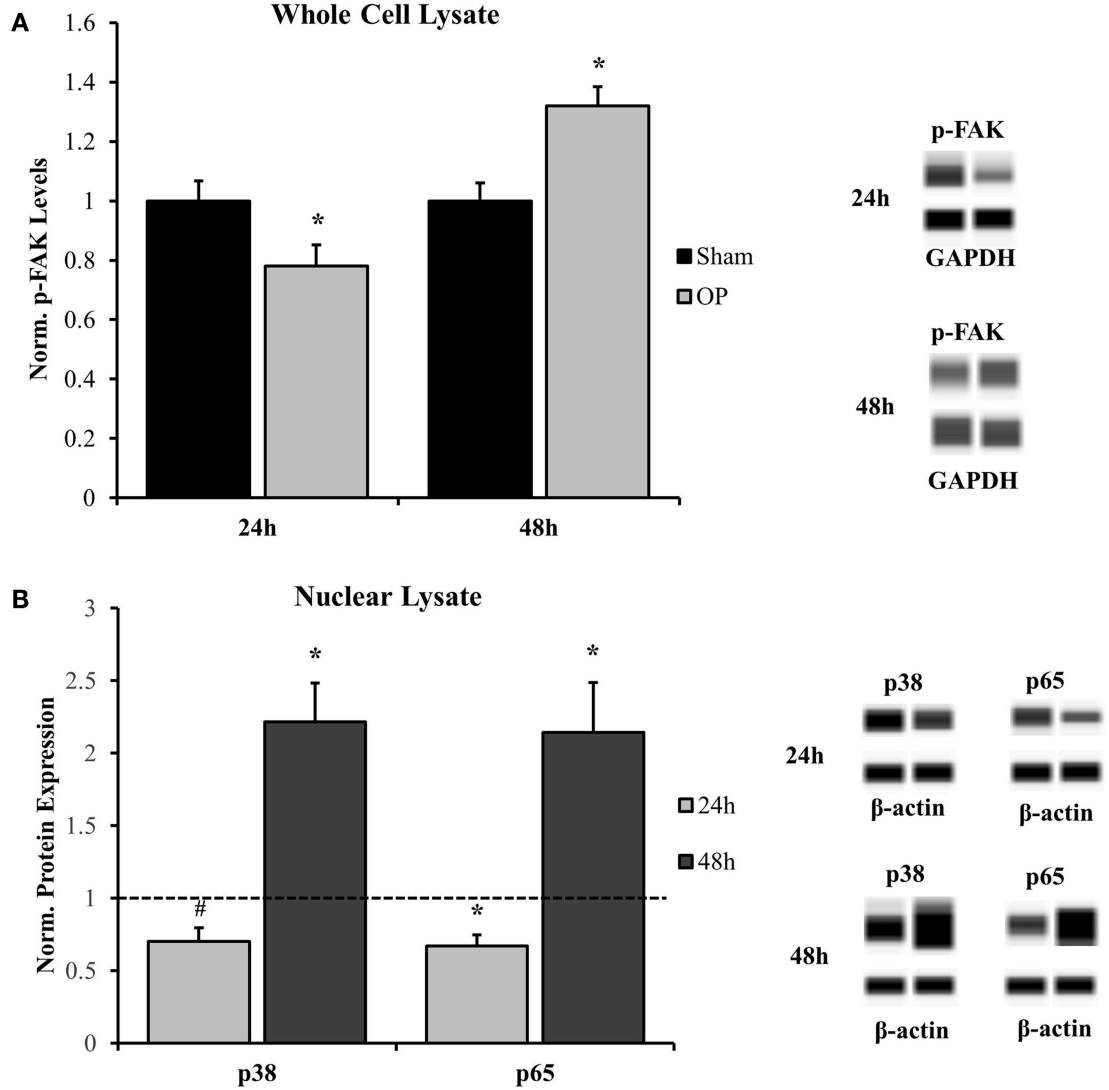
Western blot analysis for whole cell and nuclear signal transduction proteins. **(A)** Phosphorylated FAK (Y397) was significantly decreased at 24 h and significantly increased at 48 h as compared to sham. **(B)** Nuclear localization of corresponding signal transduction molecules, p38, and p65, were decreased at 24 h and increased at 48 h as compared to sham. ^*^p < 0.05, ^#^*p* = 0.056, Data are represented as mean ± SEM, whole cell: *n* = 9–10/group; nuclear: *n* = 7–9/group.

Activation of FAK by adhesion molecules and other membrane-bound proteins commonly involves phosphorylation of the molecule at tyrosine-397 (63). Results indicated that p-FAK levels were decreased at 24 h (*p* = 0.037) and increased at 48 h (*p* = 0.002) post-overpressure relative to sham (Figure 6A). Increased p-FAK corresponded to the time point where both features of classical reactivity were observed. These results implicate that this reactive profile may also be linked to dynamic changes at the membrane-level. Additionally, considering the downstream effects of FAK signaling will lead to further insights. Nuclear localization of both p38 and p65 followed a similar pattern as the FAK activation profile. A near-significant and significant decrease in localization was observed at 24 h for p38 (*p* = 0.056) and p65 (*p* = 0.020), respectively (Figure 6B). This response was shifted to a significant increase in nuclear localization for both p38 (*p* = 0.015) and p65 (*p* = 0.049) at 48 h following insult. Together, these results indicate that signaling transduction may be augmented in conjunction with increased FAK activation. The results suggest a potential pathway to relate astrocyte phenotype to functional adhesion alterations.

### NF-κB p65 Has an Increased Binding Propensity for the ICAM-1 Gene in Conjunction With Upregulated ICAM-1 Expression in Reactive Astrocytes

**Figure 7:**
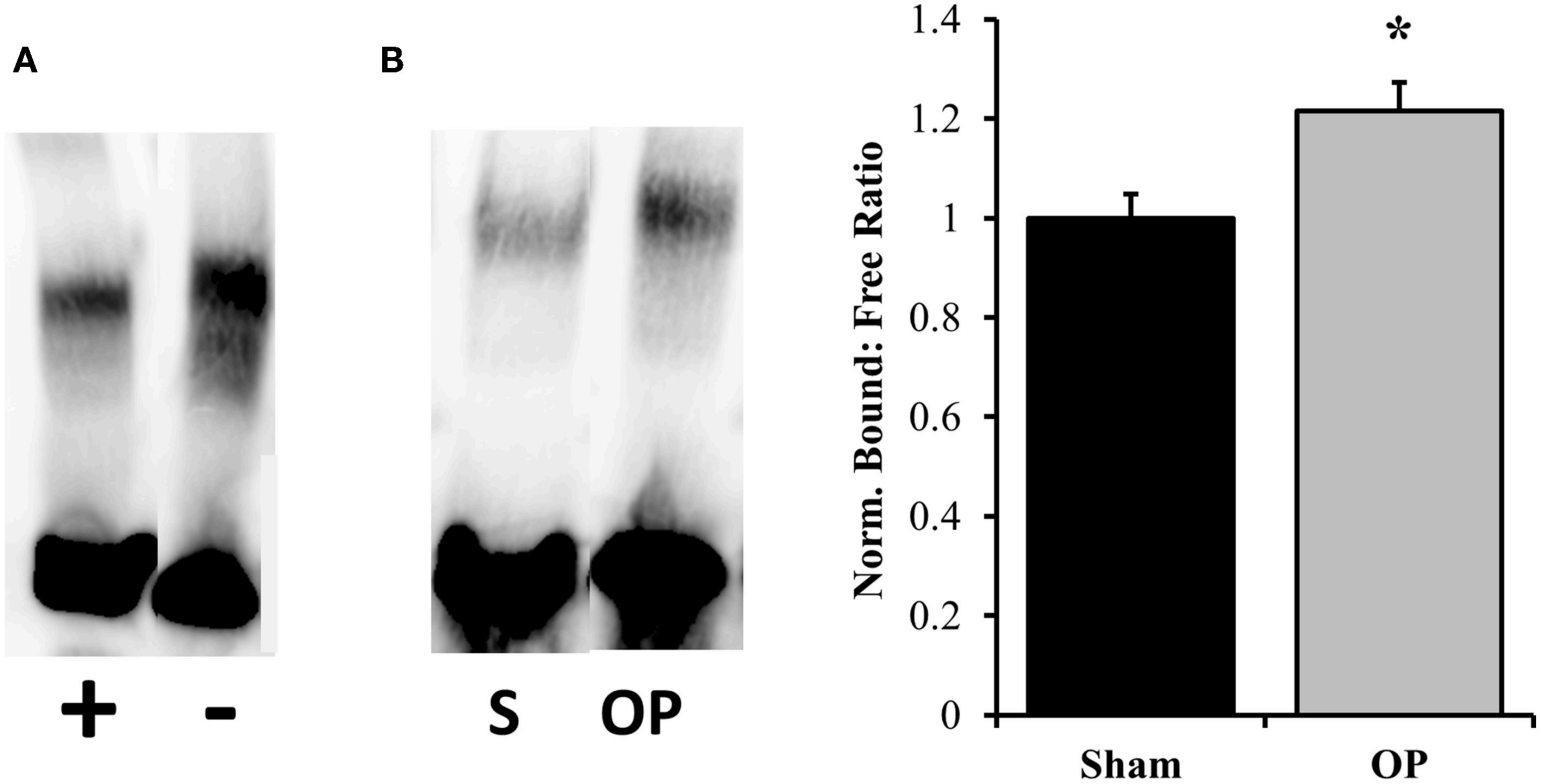
EMSA results for binding efficiency of nuclear proteins to ICAM-1 promoter sequence. **(A)** Assay optimization was conducted for specificity of protein binding to the NF-κB binding sequence of interest. The same sample was incubated with and without unlabeled DNA sequence to ensure competitive binding. The first lane contains both labeled and unlabeled DNA while the second contains only labeled. **(B)** Representative DNA: protein shifts for sham and overpressure groups are shown. Quantification of bound to free DNA determined that overpressure induced a significantly higher association of nuclear proteins with the NF-κB binding site on the ICAM-1 promoter. ^*^p < 0.05, Data are represented as mean ± SEM, *n* = 6–8/group.

**Figure 8:**
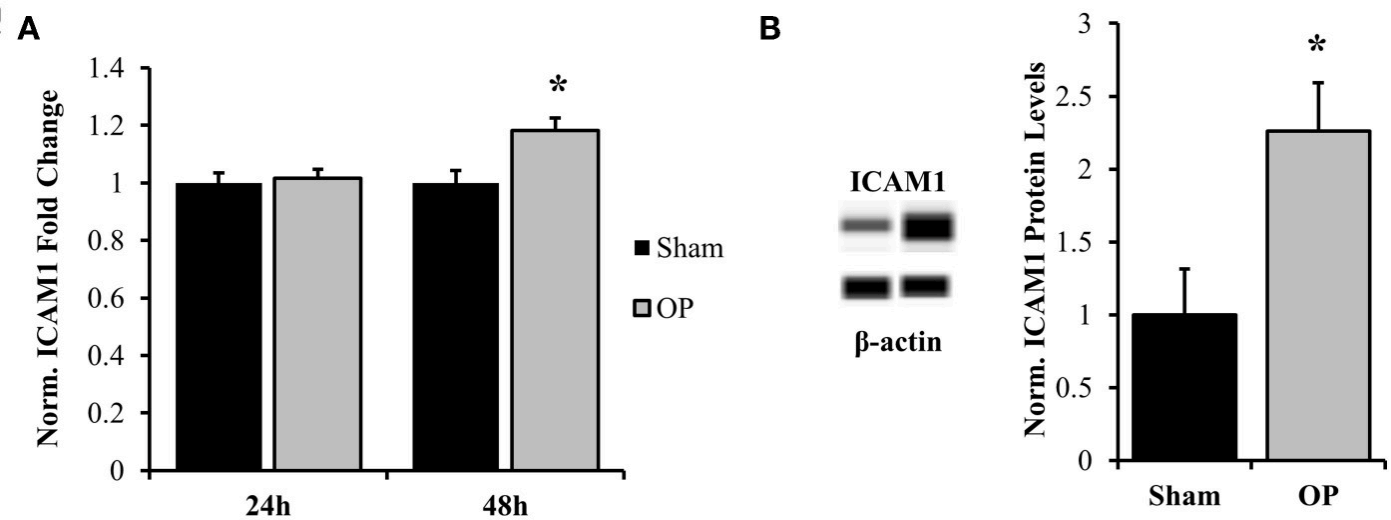
Normalized gene and protein expression of ICAM-1. **(A)** qPCR results indicated no change in mRNA expression of ICAM-1 at 24 h post-overpressure but a significant increase at 48 h as compared to sham. **(B)** Subsequent Western blot results confirmed elevated ICAM-1 protein levels at 48 h compared to sham. ^*^p < 0.05, Data are represented as mean ± SEM, gene: *n* = 7–9/group, protein: *n* = 8–9/group.

ICAM-1 is an antagonist for tight junction proteins and is expressed largely for inflammatory potentiation. Its transcription is regulated by the NF-κB pathway. To assess the potential for NF-κB localization to influence adhesion outcomes in reactive astrocytes, EMSAs were performed with nuclear extracts incubated with NF-κB-binding sequences on the ICAM-1 promoter. Optimization experiments were conducted with both biotinylated and unlabeled DNA to determine binding specificity for the given sequence (Figure 7A). A significant increase in association of nuclear proteins with the NF-κB binding sequence was observed following mechanical insult as compared to sham at 48 h (*p* = 0.013, Figure 7B). Subsequent analyses of ICAM-1 gene and protein levels were conducted to determine expression patterns. ICAM-1 gene expression was significantly increased at 48 h post-exposure as compared to sham (*p* = 0.035, Figure 8A) in conjunction with EMSA results. Gene expression was not previously increased at the 24 h time point, indicating a specificity for this time point and phenotype. The increased gene expression translated to increased protein expression at 48 h as well (*p* = 0.032, Figure 8B). These results suggest that the localized p65 had increased binding affinity for the ICAM-1 promoter in reactive astrocytes and therefore may be an important regulator of the adhesion alterations observed.

### Increased Global DNA Methylation Status Precedes Delayed Astrocyte Reactivity

**Figure 9:**
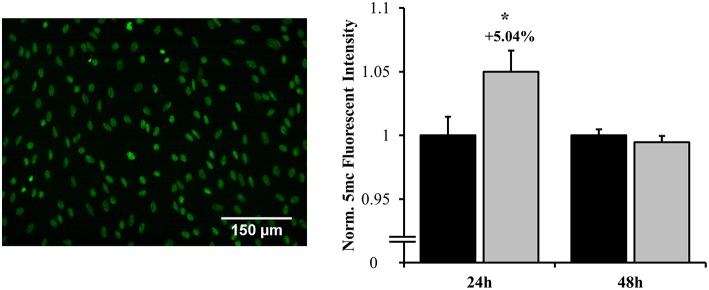
Global DNA methylation as assessed by fluorescent labeling of 5-methylcytosine. Hypermethylation occurred at 24 h post-overpressure exposure as compared to sham. No changes in methylation were observed at 48 h. ^*^p < 0.05, Data are represented as mean ± SEM, *n* = 10–12/group.

DNA methylation is an epigenetic process by which cellular transcription can be globally influenced. At 24 h, significant hypermethylation was observed (increased by 5.04%), indicating potential gene repression (*p* = 0.040) as shown in Figure 9. This response was ameliorated by 48 h, at which point no significant differences were observed between groups. This methylation pattern corresponded to both the shifts in MAPK and NF-κB localization as well as cellular phenotype that occur across this timeframe.

## Discussion

From a therapeutic standpoint, astrocytes have become an important focus in neurotrauma research because of their ability to influence many of these aspects of secondary injury sequelae (29, 64, 65). Results from the study indicate that the HOS was capable of generating a reactive response in cultured astrocytes that is phenotypically comparable to *in vivo* findings. Primary astrocytes assumed a classically-reactive response which corresponded to alterations in several classes of junctional molecules, increased PCNA expression, and mechano-activation through p-FAK. The time course of study suggested differential regulation of proliferation/repair as compared to structural reactivity in the astrocytes. PCNA is an ideal target for understanding reparative potential as it directly recruits and interacts with many DNA replication proteins (66). It has been used previously as a marker for proliferating cells, including astrocytes (67, 68). MTT results coincided PCNA expression as a significant increase in metabolic conversion was observed at 48 h. Together, these results support the assumption that isolated high-rate insult has the potential to initiate increased, although mild, reparative potential, and/or proliferation in astrocytes. It is believed that early stages of astrocyte proliferation can distinctly extend into a highly proliferative, scar-forming phenotype later after insult (69–71). Thus, better understanding of the onset of these reactive features is necessary to modulate astrocyte responses in TBI.

Additionally, molecular instigators of reactivity include broad signaling, adhesion, and structural aberrations (29, 46, 69), which are largely uncharacterized in high-rate TBI. CX43 is one such junctional molecule which participates in normal and pathological ionic and metabolite buffering as well as secondary messenger passage. It is a mechanosensitive gap junction protein with a short half-life (72–74), which may explain its early upregulation in exposed cells. CX43 is specifically expressed between astrocytes and is imperative for network communication. From *in vivo* experimentation, reactive astrocytes can display upregulated CX43 in a specific manner after TBI (75). Astrocytic CX43 has both protective and detrimental consequences after CNS insult. CX43 is mainly responsible for clearance of excitotoxic and damaging extracellular molecules after insult but also contributes to the spread of harmful signals (76–78). Evidence for significant coupling of CX43 expression with GFAP^+^ astrocytes explains one potential mechanism by which reactivity may be linked to junctional regulation (79). However, these protein networks are not physically connected and thus can respond independently in the case of injury. In general, CX43-mediated shuttling involves many types of signaling, including NF-κB activation and pro-inflammatory regulation in various pathologies (80–82) as well as regulating proliferation in other cells. Evidence exists to suggest that CX43 can lead to increased cell death after trauma because of the spread of damaging molecules (76, 78). Moreover, studies in cancer cells suggest that CX43 controls and inhibits proliferation (83, 84) and thus may be important to consider in the metabolic profiles observed.

Cellular adhesion to the external environment and to surrounding cells is another autologous regulator of mechanical stimulation within networks of cells like astrocytes. Anchoring junctions are a major group of tethering proteins that interconnect the cytoskeleton to the extracellular space. The two classes of anchoring junctions assessed in this study include cadherins and integrins. Integrins proteins form heterodimers that bind to the extracellular matrix and other extracellular adhesion molecules (85). Integrins are dynamically regulated in cells to control alterations in migration, proliferation, and adhesion (85, 86). They are important mechanosensors for normal physiological functions but have also been implicated in cellular outcomes of various CNS pathologies (57, 87–90). Integrins have been minimally explored in TBI and yet possess the potential to widely influence cellular phenotype. More specifically, integrin-β1 expression is required for astrocyte migration, stability and healing potential (88, 91–93). A few studies have implied differential expression of integrin proteins by astrocytes following mechanical trauma (94, 95). Results of this study indicate a basis for mechano-regulation of anchoring junction proteins following isolated high-rate overpressure, thus implicating them in both simple and complex cellular architectures.

This study identified that integrin-β1 and N-cadherin were upregulated in conjunction with increased structural (intermediate filament) protein expression in astrocytes. This result is supported by the fact that anchoring junctions form physical, mechano-sensing systems with intermediate filaments, which are necessary for stress modulation (96). One study showed a functional relationship between integrin-β1expression in astrocytes and structural reactivity apart from proliferation (97). Other studies indicate that integrin-β1 also directly interacts with the intermediate filament vimentin and is important for mediating proliferation in conjunction with GFAP (98, 99). There is evidence for a similar role for N-cadherin in modulating the reactive astrocyte response after traumatic insult as well (100). Together, these results indicate multiple mechanisms by which astrocyte networks may become mechano-activated in response to their altered adhesion state. It should be noted that no changes were observed in gene expression of the intermediate adaptor protein vinculin. Vinculin is widely expressed as part of the intermediate complex between the cytoskeleton and anchoring junctions. In 2D cell culture, decreased vinculin expression would support development of the migratory phenotype in cells (101). Studies have shown that expression profiles of integrin-β1 and intermediate filaments tend to be distinct in proliferating cells as compared to migrating (98).

The second portion of this study was directed at identifying autologous signal transduction activation in reactive astrocytes relative to mechano-stimulation alone. Cellular signaling mechanisms instigated by growth factor and cytokine receptors are known to influence astrocyte outcomes and often involve neuroinflammation and signals from damaged neurons (5, 28, 46). However, cell adhesion is another important component to signal transduction and may represent a critical constituent by which autologous signaling in astrocyte networks contributes to cell phenotype. In the complex TBI pathophysiology it is likely that all of these initiators contribute to astrocyte responses. Connexins, cadherins and integrins can each directly or indirectly influence ubiquitous signal transduction like MAPK and NF-κB activity via initiator proteins such as FAK (102–104). Phosphorylation of FAK can be the result of mechanical stimulation or altered cell junction properties. In the brain, FAK affects glial cell morphology and adhesion in physiological and pathological environments (105–109). One study indicated increased phosphorylated FAK (Y397) levels in reactive astrocytes following TBI (109), while another showed that inhibition of FAK phosphorylation affected reactive astrocyte migration (110). FAK is associated with initiation of adhesion signaling transduction pathways and can have a reciprocal influence on the expression and function of junctional proteins as well (111, 112). This protein kinase is phosphorylated at tyrosine-397 when the pathway is activated by integrin and other cellular adhesion stimulation at the membrane level. In response to mechanical stress, phosphorylation of FAK can often be observed very rapidly as a reaction to cell shearing (113, 114). Additionally, multiple models of the CNS insult have implicated FAK in gliosis and chronic astrocyte pathology (102, 115, 116).

**Figure 10:**
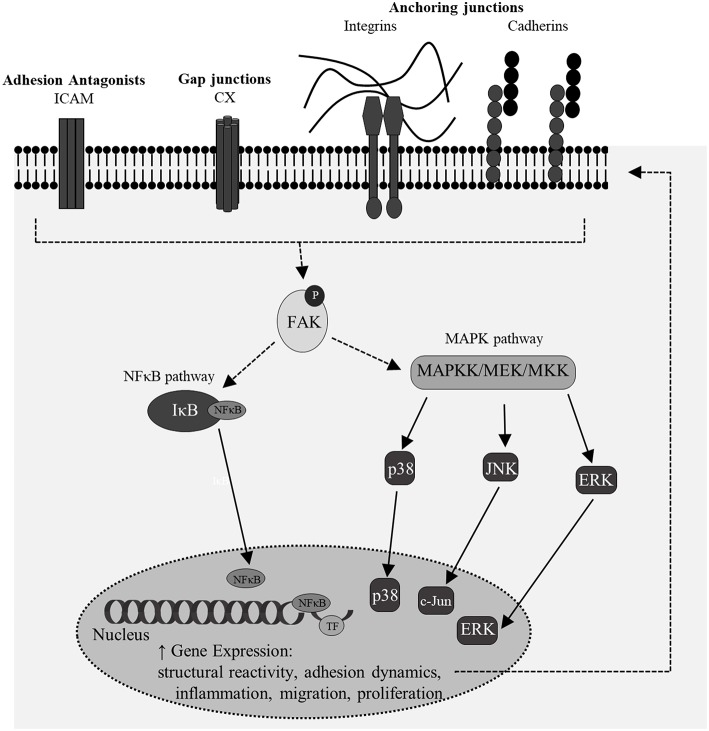
A simplistic overview of potential signaling feedback mechanisms for the regulation of junctional changes and structural reactivity in mechano-activated astrocytes. Multiple of these targets coincided with features of classical reactivity observed in this model of high-rate overpressure exposure.

Moreover, there is significant motivation to understand the molecular signature which relates initiation of dynamical adhesion signaling to downstream transduction and specific influences on gene transcription and phenotype (Figure 10). Phosphorylation of FAK can lead to subsequent activation of NF-κB and MAPK pathways, both of which influence shifts in cellular phenotype and may be important mediators in mechano-activation of astrocytes by high-rate insult. No previous studies have isolated this mechanism in astrocytes, however many have studied the mechano-regulation of NF-κB and MAPK in osteocytes, cardiomyocytes, and other uniquely activated cells (113, 117–120). Results indicated increased phosphorylation of FAK in conjunction with significantly increased nuclear localization of transcription-related molecules, p38 and p65. Upon nuclear localization, MAPK p38 can activate transcription factors, such as NF-κB p65. NF-κB p65 is a transcription factor with known binding sites on multiple adhesion genes, including ICAM-1 (121, 122).

The data showed that increased localization of p65 was accompanied by increased affinity of the transcription factor for ICAM-1 promoter, suggesting a potential pathway for regulation of adhesion molecules in reactive astrocytes. ICAM-1 is a glycoprotein that acts as an antagonist for another class of junction proteins, tight junctions. Astrocytes express ICAM-1 for pro-inflammatory potentiation as well as binding to integrin proteins on surrounding infiltrating cells (123, 124). A recent study by Lutton et al showed that increased ICAM-1 expression in a mouse cortical impact model was associated with vascular endothelial cells and activated glial cells, likely astrocytes (125). The study also inhibited ICAM-1 expression to decrease oxidative stress, blood brain barrier permeability and microglial co-activation. Other studies have reported ICAM-1 associated with region-specific glial activation in various CNS pathologies (126, 127), however, this is the first report that links astrocytic ICAM-1 regulation to high-rate mechanical insult. The NF-κB pathway is important in mediating neuroinflammation, structural phenotype and cell survival. Additionally, MAPK and NF-κB molecules influence the activation of other transcription factors in the nucleus for broader impacts on cellular phenotype. Co-activation of transcription factors may provide the molecular link between structural reactivity, proliferative phenotype, and adhesion-mediated dynamics. Future studies should exploit these pathways to understand their specific contributions to outcomes related to astrocyte reactivity.

Lastly, signal transduction mechanisms can influence and are influenced by DNA methylation status. Hypermethylation status corresponds to patterns of gene repression, while the opposite is true for hypomethylation. Previously, DNA methylation changes have been observed in blast neurotrauma (17, 128). Results of this study suggest that DNA hypermethylation may be an important mediator in the delay of structural reactivity responses observed here and in *in vivo* studies of blast neurotrauma. It is also possible that CX43 overexpression and DNA methylation may co-exist to mediate proliferative responses and may be potentially linked to metabolic and redox state of the cells, but much more work is needed to understand this relationship. Future work will exploit methods to understand more specific DNA methylation patterns related to the features of reactivity obtained in this study.

It is necessary to consider certain limitations of this model. The presented *in vitro* model is a 2D, astrocyte-only system and therefore may not recapitulate certain aspects of cellular focal adhesion as found in the native brain tissue. This will be especially important to consider the non-mechanical instigators of astrocyte reactivity, notably inflammatory and metabolic stress mechanisms, and how they interact with mechanically-derived signals to cause persistent activation. Additionally, adhesion organization and activation of signaling molecules such as FAK are different in 3D as compared to 2D (129, 130). This relationship should be considered in future studies evaluating expression of these proteins by cells within a heterogeneous ECM. Cellular adhesion formation and migration is spatially dependent on extracellular architecture. However, the purpose of this study was to establish the ability of high-rate overpressure to induce both classical reactivity and dysregulated expression of adhesion molecules simultaneously. Further studies will be necessary to understand the functionality of these adhesion proteins as this work only assesses their regulation and expression profiles in reactive astrocytes. For instance, in 2D, many of the functions for integrin proteins are conserved, including anchorage and polarity, but 3D studies will be necessary to assess focal adhesion formation (131). Secondly, the time course and persistence of astrocyte activation may be influenced by the presence of other cells and environmental stimuli (28, 46). Future studies should also address responses in specific astrocyte populations within the brain (i.e., hippocampal) to assess changes in behaviors associated with these particular areas.

This study advances understanding of primary astrocyte response to high-rate insult and identifies multiple molecular targets for aberrant astrocyte adhesion. The purpose of this study was to develop an *in vitro* model to characterize primary cell response to high-rate compressive overpressure. In addition, it creates a platform to further study adhesion dynamics in advanced 3D cell culture models. Altogether, the presented results have provided progress toward both fundamental understanding of initiating mechanisms for astrocyte reactivity but also into novel considerations for therapeutic modulation of astrocytes to improve TBI outcomes. This correlative study established specific profiles of adhesion/junctional protein regulation which correspond to aspects of astrocyte reactivity. Moreover, results have identified novel targets at multiple levels within signaling mechanisms for astrocyte mechano-activation. Future studies will specifically intervene in pathways associated with integrin and other cell adhesion molecules to determine their influences on astrocyte network function with the goal of interventional modulation of reactive astrocytes for improved brain repair.

## Author Contributions

PV designed and optimized the *in vitro* overpressure device used in this study. NH conducted cell culture, overpressure exposure, and molecular analyses. PV and NH contributed to written manuscript.

### Conflict of Interest Statement

The authors declare that the research was conducted in the absence of any commercial or financial relationships that could be construed as a potential conflict of interest.

## Supplementary Material

Figure S1 Representative Western blot data from the Wes. Shown are two samples (overpressure and sham) with protein target PCNA (37 kDa) and loading control β-actin (48 kDa). All analyses were conducted using areas quantified at set exposure times as acquired by the Wes software, Compass.
