# Supplementary figures and images for: Astrocyte Mechano-Activation by High-Rate Overpressure Involves Alterations in Structural and Junctional Proteins
Source: Front Neurol. 2019 Feb 22;10:99. doi: 10.3389/fneur.2019.00099 (PMC6395392; doi:10.3389/fneur.2019.00099)

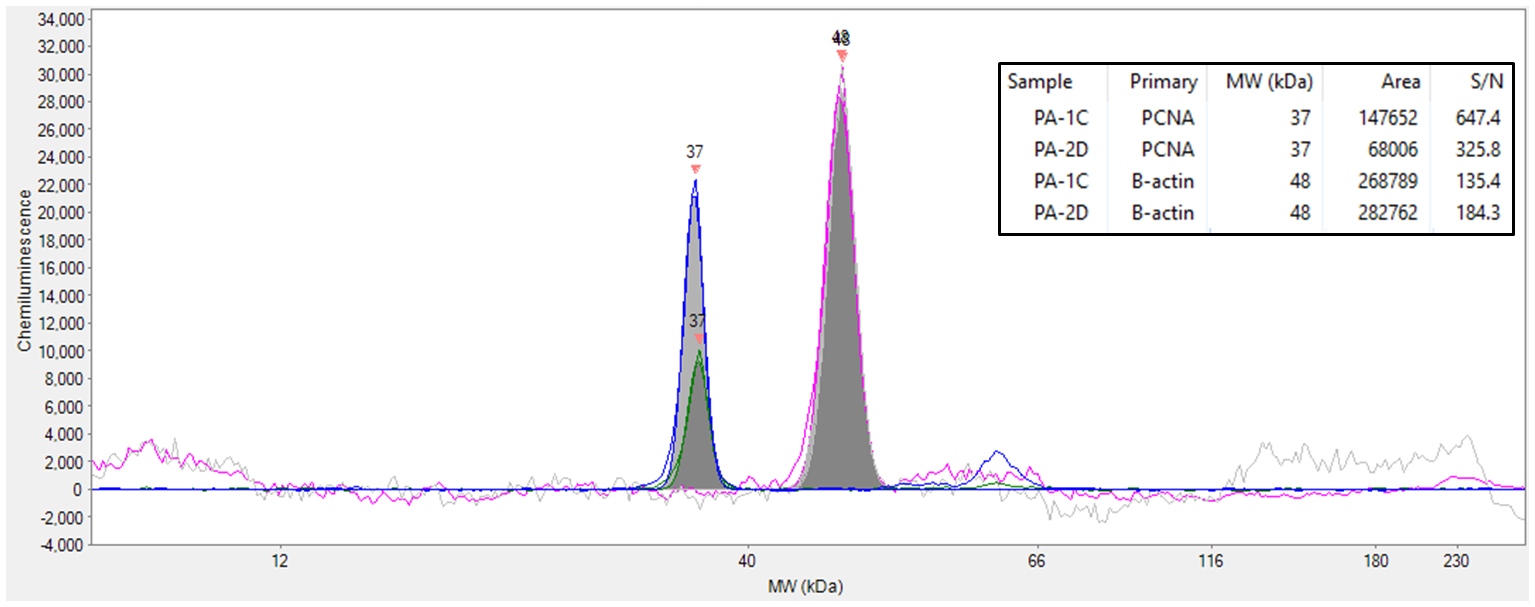

Supplement: Figure S1 — Representative Western blot data from the Wes. Shown are two samples (overpressure and sham) with protein target PCNA (37 kDa) and loading control β-actin (48 kDa). All analyses were conducted using areas quantified at set exposure times as acquired by the Wes software, Compass. [file Image_1.TIF]
